# Bioactive Effects of Plectranthus amboinicus Extract Using Microwave Techniques and Its Value Addition in Cosmeceutical Products

**DOI:** 10.12688/f1000research.165030.2

**Published:** 2025-10-13

**Authors:** Chananan Chinnahong, Warut U-Kong, Thiravat Rattanapot, Chetsalit Hongnueng, Doungporn Amornlerdpison

**Affiliations:** 1 Interdisciplinary Agriculture Program, Faculty of Agricultural Production,, Maejo University, Nong Han, Chiang Mai, Thailand; 2 Center of Excellence in Agricultural Innovation for Graduate Entrepreneur, Maejo University, Nong Han, Chiang Mai, Thailand; 3 Faculty of Fisheries Technology and Aquatic Resources, Maejo University, Nong Han, Chiang Mai, Thailand

**Keywords:** Plectranthus amboinicus, microwave-assisted extraction, antioxidant activity, antimicrobial activity, anti-inflammatory activity

## Abstract

**Background:**

*Plectranthus amboinicus* is an aromatic herb known for its medicinal properties and is increasingly explored for cosmetic applications. Its bioactive compounds possess antioxidant, antimicrobial, and anti-inflammatory properties, making it a promising candidate for multifunctional skincare formulations.

**Methods:**

This study investigated the extraction of bioactive compounds from *P. amboinicus* leaves using microwave-assisted ethanol extraction. Two drying techniques—tray drying and freeze-drying—were compared to evaluate their impact on the extraction efficiency. The optimal extract (PF15), prepared using 15-minute microwave extraction and freeze-drying, was selected for further analysis. Bioactive content was assessed through quantification of caffeic acid, total phenolic content, and antioxidant activity via the DPPH assay. The antimicrobial activity of PF15 was tested against *Staphylococcus aureus*, *Staphylococcus epidermidis*, and *Cutibacterium acnes.* Anti-inflammatory potential of PF15 was evaluated in LPS-stimulated human THP-1 macrophages by measuring cytokine production.

**Results:**

The PF15 extract yielded the highest levels of bioactive compounds and demonstrated strong antioxidant activity. It exhibited significant antimicrobial effects against all tested skin pathogens. In the anti-inflammatory assay, PF15 significantly decreased pro-inflammatory cytokines (TNF-α, IL-1β, and IL-6) while upregulating the anti-inflammatory cytokine IL-10. The extract was formulated into a topical cream, which underwent accelerated stability testing over six heat-cool cycles. The cream remained stable with no signs of phase separation, discoloration, odor change, or microbial contamination, and maintained a pH of 5.5.

**Conclusions:**

The PF15 extract of *P. amboinicus* demonstrates potent antioxidant, antimicrobial, and anti-inflammatory properties. Its successful incorporation into a stable cream formulation supports its potential as a multifunctional active ingredient in skincare products. These findings highlight *P. amboinicus* as a valuable natural source for the development of cosmetic formulations targeting oxidative stress, microbial infection, and inflammation.

## Introduction

The global herbal cosmetic market has experienced consistent growth in recent years, driven by increasing consumer demand for natural and organic products. This trend reflects heightened awareness of the adverse effects associated with synthetic chemicals commonly used in cosmetic formulations, including allergic reactions, skin irritation, and potential long-term health risks. According to Custom Market Insights, ^1^ the global market value of herbal beauty products—which encompasses skincare, haircare, and fragrances for both men and women—was estimated at USD 78.9 billion in 2023 and is projected to reach USD 150.2 billion by 2032, with a compound annual growth rate (CAGR) of 6.5%.

The application of plant extracts in cosmetic products has surged due to their diverse biological activities, such as antioxidant, anti-inflammatory, UV-protective, anti-aging, and anti-acne effects. ^2^ These benefits, coupled with growing concerns over the safety of synthetic ingredients, have prompted a shift in consumer preference towards natural alternatives. Medicinal plants offer a promising and safer source of bioactive compounds for use in cosmetic formulations. In the context of Thailand, the integration of native medicinal plants into cosmetic development presents an opportunity to enhance the value and global competitiveness of local herbal resources. Various plant parts—including leaves, flowers, fruits, stems, and roots—are known to exhibit a wide range of bioactivities, including antibacterial, antifungal, and yeast-inhibitory effects, along with properties that support skin nourishment and restoration. ^3^

*Plectranthus amboinicus*, a perennial herb indigenous to Southeast Asia, has been widely utilized in traditional culinary practices and herbal medicine. Its leaves are rich in numerous bioactive constituents, such as essential oils (e.g., carvacrol, thymol, γ-terpinene) and phenolic compounds (e.g., caffeic acid, quercetin, ursolic acid, and rosmarinic acid), which exhibit potent antioxidant, antimicrobial, and anti-inflammatory properties. Additionally, these compounds have demonstrated other pharmacological activities, including anticancer potential. ^4, 5^ A recent study by Ref. 6 demonstrated the antimicrobial efficacy of *P. amboinicus* leaf extract against pathogens such as *Staphylococcus aureus*, *Escherichia coli*, *Pseudomonas aeruginosa*, *Candida albicans*, and the acne-associated bacterium *Cutibacterium acnes*, reinforcing its potential application in cosmetic products.

Various techniques have been employed to extract bioactive compounds from plants, including maceration, Soxhlet extraction, supercritical CO _2_ extraction, hydrothermal processing, and ultrasonic-assisted extraction. Despite their widespread use, these conventional methods often involve extended processing times, low extraction efficiency, and high operational costs. ^7^ Recently, microwave-assisted extraction (MAE) using 50% ethanol has emerged as a promising alternative. This technique offers several advantages, including shorter extraction time, higher yield and concentration of bioactive compounds, and reduced solvent consumption. ^8^ Moreover, MAE maintains the integrity and efficacy of extracts while ensuring safety and environmental sustainability, making it well-suited for industrial and commercial-scale applications. ^9, 10^

Accordingly, the present study aimed to evaluate the extraction of bioactive compounds from *P. amboinicus* leaves using microwave-assisted ethanol extraction. This was followed by a comparative evaluation of two drying techniques and the assessment of the extract’s antioxidant, antimicrobial, and anti-inflammatory activities. The final objective was to incorporate the extract into a prototype cosmetic formulation, such as an anti-acne or anti-inflammatory cream. The outcomes of this research are expected to enhance the commercial value of Thai medicinal herbs and promote the sustainable cultivation of *P. amboinicus*, thereby generating economic benefits for local communities.

## Materials and methods

### Extraction process optimization for *Plectranthus amboinicus* leaves

**Sample collection and preparation**

*Plectranthus amboinicus* leaves were collected from the Maejo area, San Sai District, Chiang Mai Province, Thailand. The leaves were washed with distilled water and dried in a hot air oven at 60 °C for 24 hours. Once dried, they were ground into a fine powder. Three separate 20-gram portions of the powdered leaves were weighed and placed into round-bottom flasks for extraction.

**Microwave-assisted extraction procedure**

Bioactive compounds were extracted using microwave-assisted extraction (MAE) at a power of 450 watts, using 200 mL of 50% ethanol as the solvent at temperature 40°C. The 50% ethanol solution was prepared using a 1:1 mixture of ethanol and deionized water. The extraction was conducted at three different time intervals: 10, 15, and 20 minutes. ^11^ The extracts were filtered using Whatman No.1 filter paper, and the solvents were partially evaporated under reduced pressure using a rotary evaporator. Extracts were then stored at −40 °C for further analysis.

**Drying methods and yield determination**

The filtered extracts were subjected to two drying techniques: freeze-drying (PF) and tray-drying (PT). The percentage yield (% yield) was calculated using the formula:

%Yield=(Weight of Dried Extract/Weight of Starting Material)×100

### Chemical characterization

**Caffeic acid quantification by HPLC**

Extracts from MAE (PT10, PT15, PT20, PF10, PF15, PF20) were analyzed for caffeic acid content. It was the most abundant phenolic identified in preliminary HPLC profiling. The standard of caffeic acid was used to construct the calibration curve. Each 0.05 g sample was dissolved in ethanol and diluted to 50 mL. Solutions were filtered through a 0.45 μm membrane filter and placed into 2 mL vials. High-performance liquid chromatography (HPLC) was used for quantification. Conditions are listed in Table 1.

**Table 1.** HPLC conditions (FLEXAR™ LC System, PerkinElmer).

Condition	
Column	Brownlee Analytical C18, 150 × 4.60 mm, 5.0 μm
Mobile Phase	(A) 0.1% Phosphoric acid in water, (B) Methanol (80:20)
Flow Rate	0.3 mL/min
Injection Temperature	40 °C
Detector	Photodiode array detector (PDA), 275 nm
Injection Volume	5 μL
Mode of elution	Isocratic elution

**Total Phenolic Content (TPC) determination**

TPC was measured using the Folin–Ciocalteu method on PF10, PF15, and PF20 samples. Extracts (200 μL) were mixed with 1,000 μL of Folin–Ciocalteu reagent and 800 μL of 7.5% sodium carbonate. After incubation at room temperature for 60 minutes, absorbance was measured at 765 nm. A gallic acid standard curve (16–250 mg/L) was used to calculate phenolic content, reported as mg gallic acid equivalents per gram (mg GAE/g extract). ^12, 13^

### Biological activity assessment

**Antioxidant activity (DPPH assay)**

The antioxidant activity of PF10, PF15, and PF20 was assessed using the DPPH (2,2-diphenyl-1-picrylhydrazyl) radical scavenging method with some modification. ^14^ Various concentrations of extract (0.125–4 mg/mL) and gallic acid (0.01-0.05 mg/mL) were tested. After mixing 50 μL of each sample with 100 μL DPPH (2,000 μM), the reaction was incubated in darkness for 30 minutes. Absorbance was read at 517 nm.

Scavenging activity (%) was calculated as:

%DPPH=[(A_control−A_sample)/A_control]×100

The IC _50_ value, indicating the concentration required to scavenge 50% of DPPH radicals, was used to compare antioxidant potential.

**Antimicrobial activity**

***Bacterial preparation***

PF15 was tested against *Staphylococcus aureus* (ATCC 25932), *Staphylococcus epidermidis* (ATCC 14990), and *Cutibacterium acnes* (ATCC 6919). Bacteria were cultured in nutrient broth at 37 °C for 12–18 hours and adjusted to a 0.5 McFarland standard, giving an approximate cell density of 1.5 × 10 ^8^ CFU/mL.

***Extract preparation for antimicrobial testing***

A 1,000 mg/mL stock solution of PF15 was prepared in sterile distilled water, filtered through a 0.2 μm membrane and used for antimicrobial testing.

***Disc diffusion assay***

Sterile discs were loaded with 10 μL of extract, dried, and placed on agar plates seeded with test bacteria. Sterile water and ampicillin (10 μg/disc) served as negative and positive controls. Plates were incubated at 37 °C for 24 hours and inhibition zones were measured. ^15^

***MIC determination (Broth microdilution)***

PF15 was serially diluted in 96-well plates (1,000–0.48 mg/mL). Wells were inoculated with bacteria, and plates incubated at 37 °C for 24 hours. The MIC (Minimal Inhibitory Concentration **)** was the lowest concentration with no visible bacterial growth. ^15^

***MBC determination (Drop plate method)***

Aliquots from each MIC well were plated on nutrient agar. After incubation, the MBC (Minimal Bactericidal Concentration was the lowest concentration at which no bacterial colonies formed. ^15^

**Anti-inflammatory activity assay**

***THP-1 Cell culture and differentiation***

THP-1 monocytes (ATCC TIB-202) were cultured in RPMI-1640 medium with 10% FBS and 1% antibiotics. Differentiation into macrophages was induced using 50 ng/mL PMA for 48 hours, followed by 24 hours in fresh medium. ^16^

***Cell viability assay***

Differentiated cells were treated with PF15 extract (0–200 μg/mL) and 0.5 μg/mL LPS (lipopolysaccharides) for 24 hours. PrestoBlue ^®^ reagent was added, and absorbance was measured at 570 nm to assess cell viability. ^17^

**Cytokine measurement by ELISA**

Cells were treated with PF15 (12, 25, and 50 μg/mL) along with LPS. Supernatants were collected after 24 hours and analyzed for TNF-α, IL-1β, IL-6, and IL-10 using ELISA kits (Thermo Fisher). Results represent means ± standard deviation (SD) from triplicate experiments.

### Product development and evaluation

**Cream Formulation with PF15**

An anti-acne cream was formulated using the MIC concentration of PF15. The extract was incorporated into the cream formulation at a concentration of 0.8% w/w, ensuring antimicrobial efficacy. The aqueous phase containing PF15 was heated to 70 °C, combined with the oil phase, and emulsified for 30 minutes. The cream was then cooled to 35–40 °C, mixed until homogeneous, and stored for further analysis. A topical cream was developed by incorporating the PF15 extract into a water-in-oil emulsion system. The detailed composition of the cream is presented in Table 2.

**Table 2.** The composition of the PF15 cream.

Component	Concentration (% w/w)
Cream base 6065	6.0
Cetyl alcohol	1.0
Mineral oil	4.0
Isopropyl myristate	4.0
Tocopherol	1.0
PF15	0.8
Glycerin	1.0
Distilled water	82.2

**Stability and safety evaluation**

***Accelerated stability testing***

The cream underwent 6 thermal cycles (4 °C for 24 h and 45 °C for 24 h) to assess changes in pH, odor, color, texture, and phase separation. ^18^

***Antimicrobial activity of the final product***

Using the disc diffusion method, antimicrobial activity was evaluated against *S. aureus*, *S. epidermidis*, and *C. acnes.* Cream without extract served as a negative control.

***Microbial contamination testing***

**Aerobic Plate Count (APC)**

Cream samples were diluted and plated on plate count agar. Colonies were counted after 24 hours at 37 °C, reported as CFU/g.

**Yeast and mold contamination**

Samples were plated on Dichloran Rose Bengal Chloramphenicol agar after serial dilution. Colonies were counted after 24 hours to determine CFU/g.

***Escherichia coli* detection**

Diluted samples were cultured in Lauryl Tryptose broth and incubated. Gas formation indicated presumptive *E. coli.* Confirmation was done using EC Broth and MPN methodology.

***Staphylococcus aureus* detection**

Samples were diluted and plated on Mannitol Salt agar. Colony morphology and color change were used for identification. CFU/g was reported. ^19^

***Salmonella* spp. detection**

Samples were pre-enriched in Tryptone Soya broth, streaked onto SS Agar, and incubated for 24 hours. Growth consistent with *Salmonella* spp. was recorded as “present” or “absent” in 25 g of sample. ^20^

### Statistical analysis

All results are presented as mean ± SD. Statistical significance was assessed using one-way ANOVA, followed by Duncan’s Multiple Range Test. A *p*-value < 0.05 was considered significant. Data analysis was conducted using GraphPad Prism version 9.0.0.121.

## Results and Discussion

### Yield of extraction and physical characteristics of *Plectranthus amboinicus* extracts

**Figure 1.**
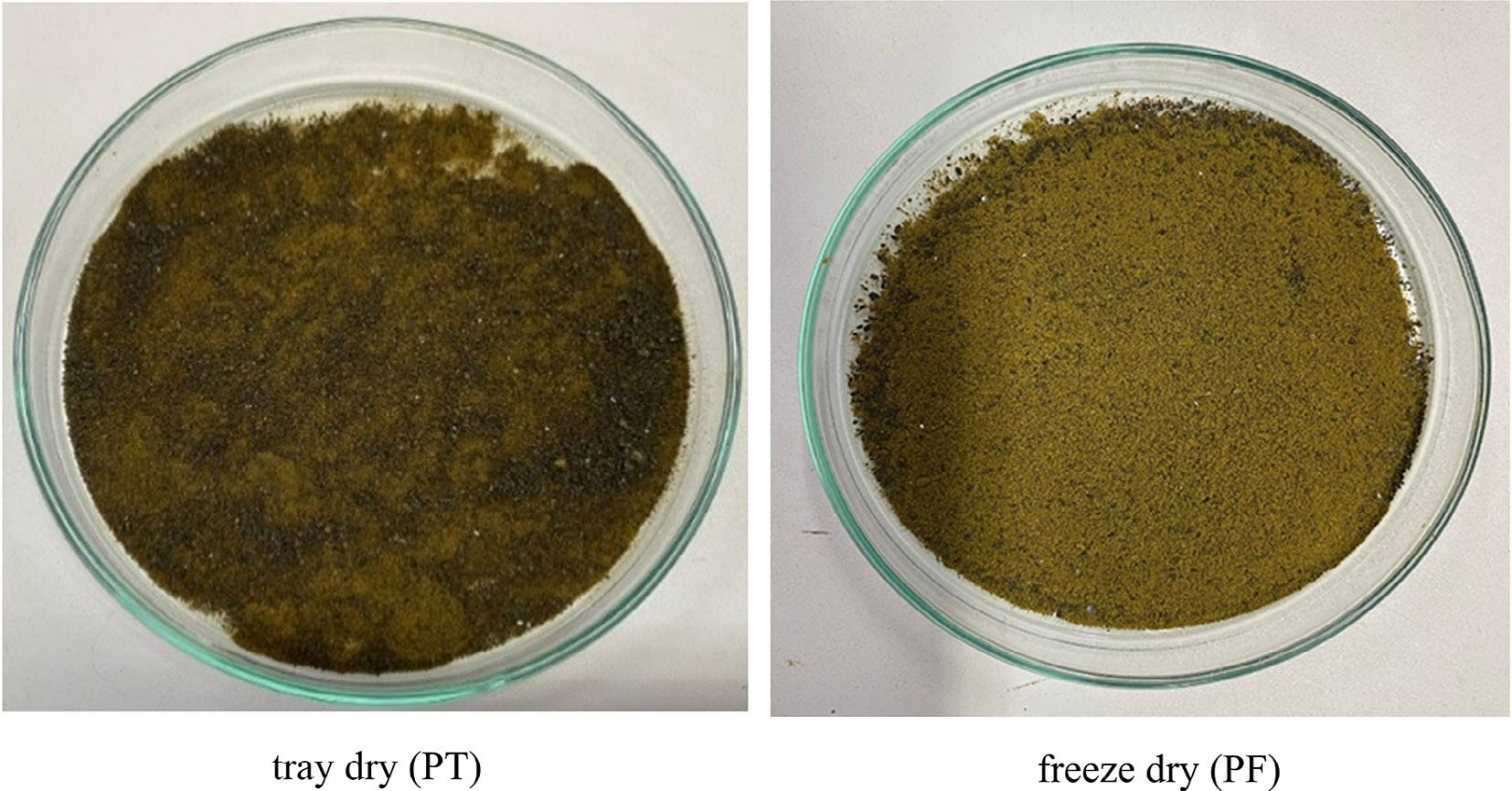
Physical characteristics of *P. amboinicus* extracts obtained by tray drying and freeze-drying.

**Figure 2.**
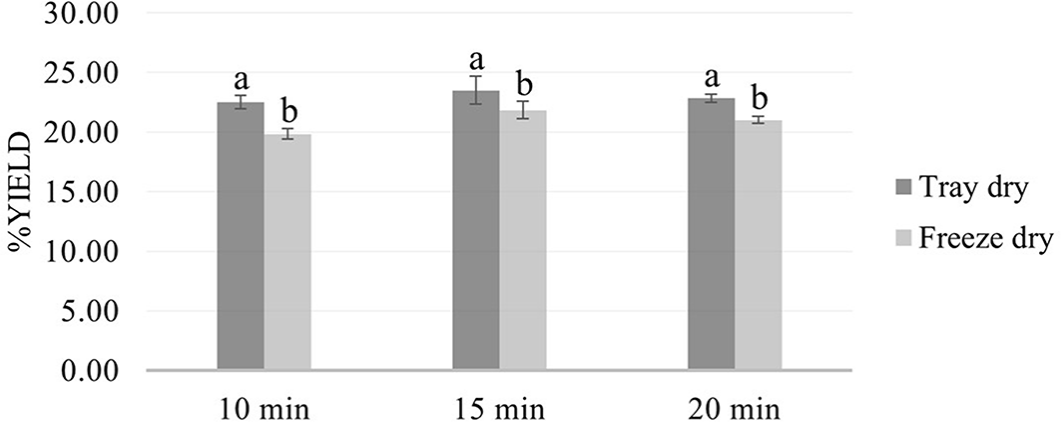
Comparative yield of *P. amboinicus* extracts by drying method and extraction time.

Microwave-assisted extraction (MAE) using 50% ethanol for 10, 15, and 20 minutes, followed by either tray drying (PT10, PT15, PT20) or freeze-drying (PF10, PF15, PF20), yielded extracts with distinct physical characteristics and varying efficiencies. Tray-dried extracts produced yields of 22.4, 23.5, and 22.5% w/w dried weight, respectively, demonstrating consistent extraction efficiency across the time intervals. These values are in alignment with the findings ^21^ which reported diminishing returns with extended extraction beyond optimal durations. The resulting tray-dried powders were dark green in color, indicating good retention of chlorophyll, flavonoids, and phenolic compounds ( Figure 1). Conversely, freeze-dried extracts yielded slightly lower quantities—19.8, 21.8, and 21.0 g for PF10, PF15, and PF20, respectively—yet retained a fine, homogeneous powder with vibrant green coloration. Despite lower yields, freeze-drying was effective in preserving thermolabile bioactives and improving powder texture. These visual differences in texture and color between drying methods ( Figure 2) highlight the critical influence of post-extraction drying on the final product characteristics, with freeze-drying offering superior preservation at the expense of yield. ^22^

### Caffeic acid content analysis

**Tray-dried extracts (PT)**

**Figure 3.**
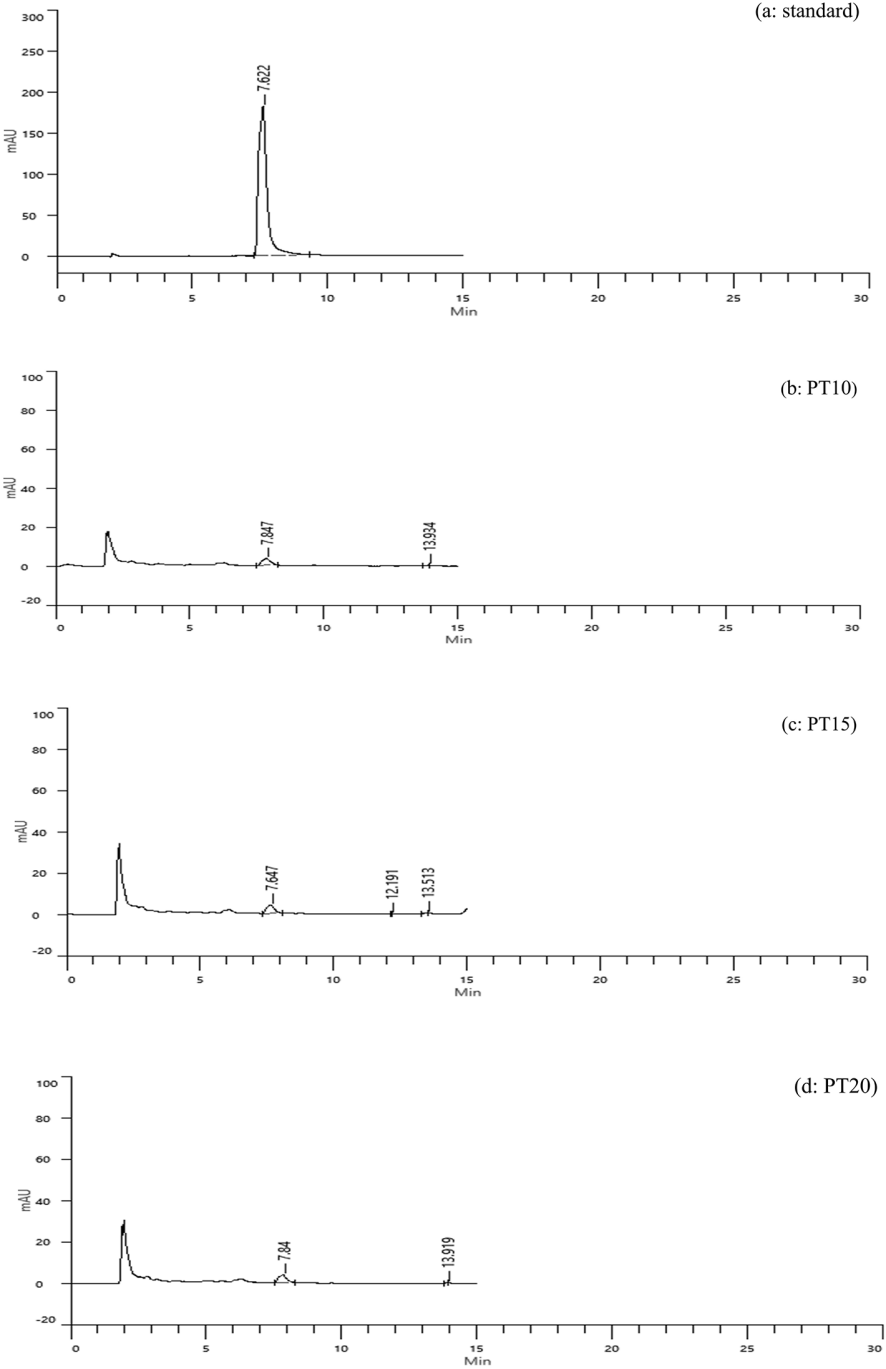
HPLC chromatograms of caffeic acid standard and tray-dried samples.

High-performance liquid chromatography (HPLC) analysis confirmed the presence of caffeic acid in all tray-dried extracts. The retention times observed for PT10, PT15, and PT20 were 7.847, 7.647, and 7.84 minutes, respectively, with corresponding concentrations of 1.04, 1.07, and 1.03 mg/g ( Figure 3b-d). These results reflect consistent caffeic acid retention across varying extraction times, with PT15 slightly outperforming others. However, the overall concentration remained modest, suggesting that tray drying may limit phenolic preservation.

**Freeze-dried extracts (PF)**

**Figure 4.**
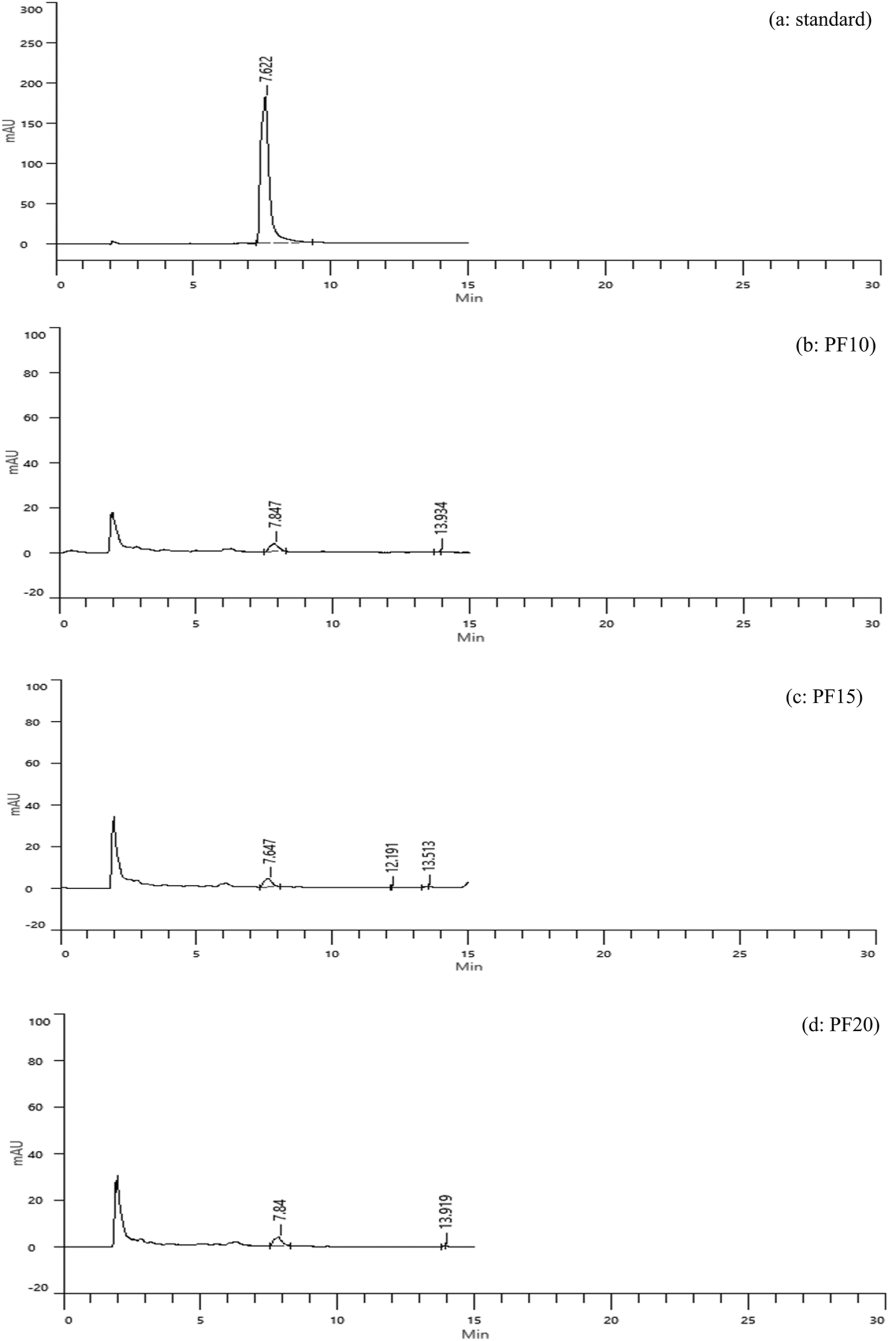
HPLC chromatograms of caffeic acid standard and freeze-dried samples.

Caffeic acid content in freeze-dried extracts was significantly higher: 3.15, 3.33, and 3.29 mg/g for PF10, PF15, and PF20, respectively ( Figure 4b-d). Retention times were consistent with the standard, confirming accurate identification. PF15 exhibited the highest caffeic acid content, indicating that 15 minutes of MAE under freeze-drying conditions is high phenolic preservation. The increased levels of caffeic acid in PF samples may be attributed to the lower thermal stress during drying, which minimizes degradation of sensitive compounds. ^21^

**Figure 5.**
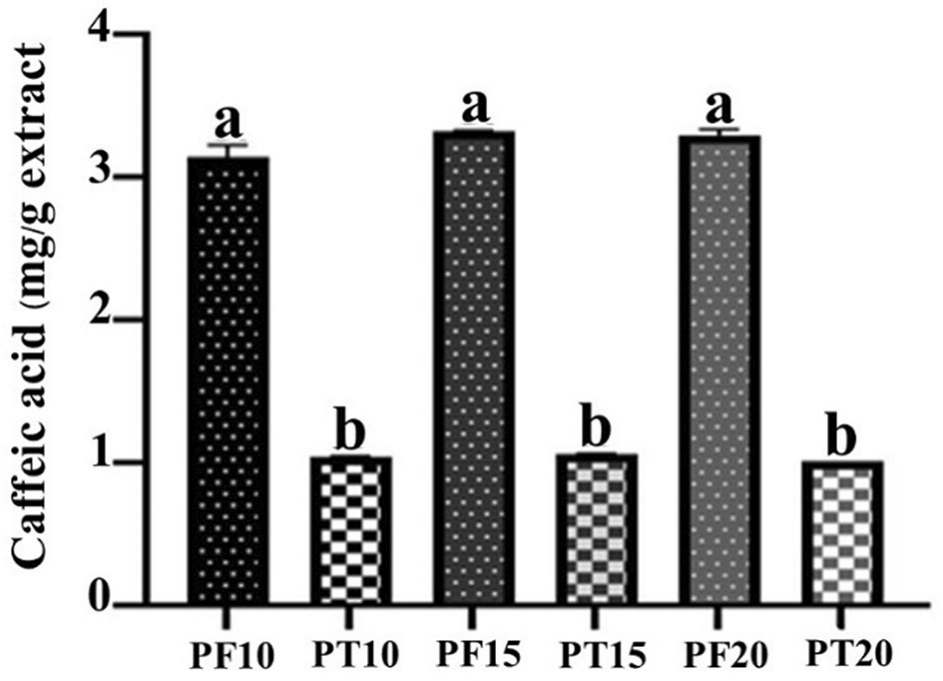
Quantitative comparison of caffeic acid content in PT vs. PF samples at different extraction times.

The comparative analysis ( Figure 5) clearly illustrates that freeze-drying consistently outperformed tray drying in preserving caffeic acid content across all extraction durations. This emphasizes the role of gentle drying techniques in maintaining the chemical integrity of phytochemicals, supporting the use of freeze-drying for applications targeting high bioactive potency.

### Total Phenolic Content (TPC) evaluation

**Figure 6.**
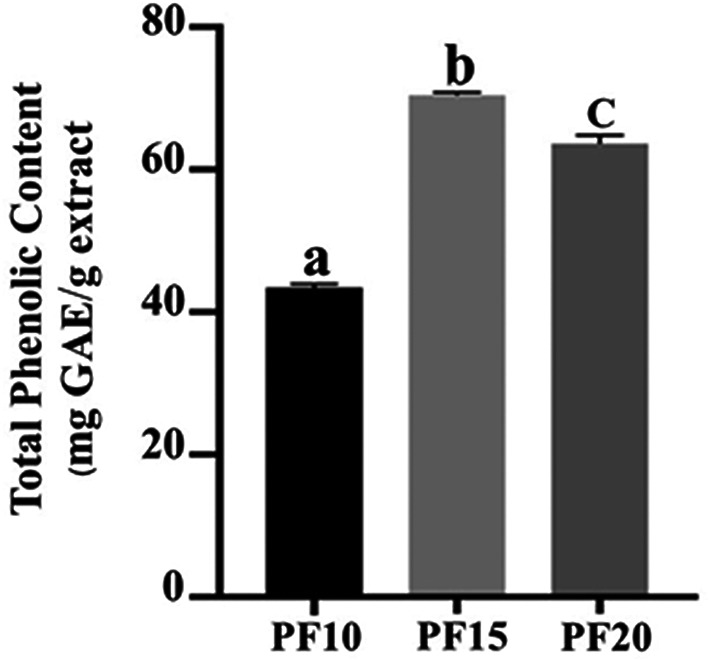
Total phenolic content in PF10, PF15, and PF20 samples.

The TPC was highest in PF15, recorded at 70.46 ± 0.49 mg GAE/g extract, followed by PF10 and PF20. The differences were statistically significant ( *p* < 0.05), indicating that extraction time had a measurable impact on phenolic recovery ( Figure 6). Notably, the 15-minute extraction (PF15) demonstrated high yield and stability, aligning with earlier research indicating that mid-range MAE durations improve phenolic extraction without inducing degradation. ^23, 21^ These results collectively underscore the effectiveness of microwave-assisted extraction at 15 minutes in conjunction with freeze-drying as the most favorable combination for preserving caffeic acid and phenolic content in *P. amboinicus* leaf extracts. The application of this selected method is particularly advantageous in the development of high-performance cosmeceutical products where antioxidant activity is desired.

### Biological activities of *Plectranthus amboinicus* leaf extracts (PF15)

**Antioxidant activity**

**Figure 7.**
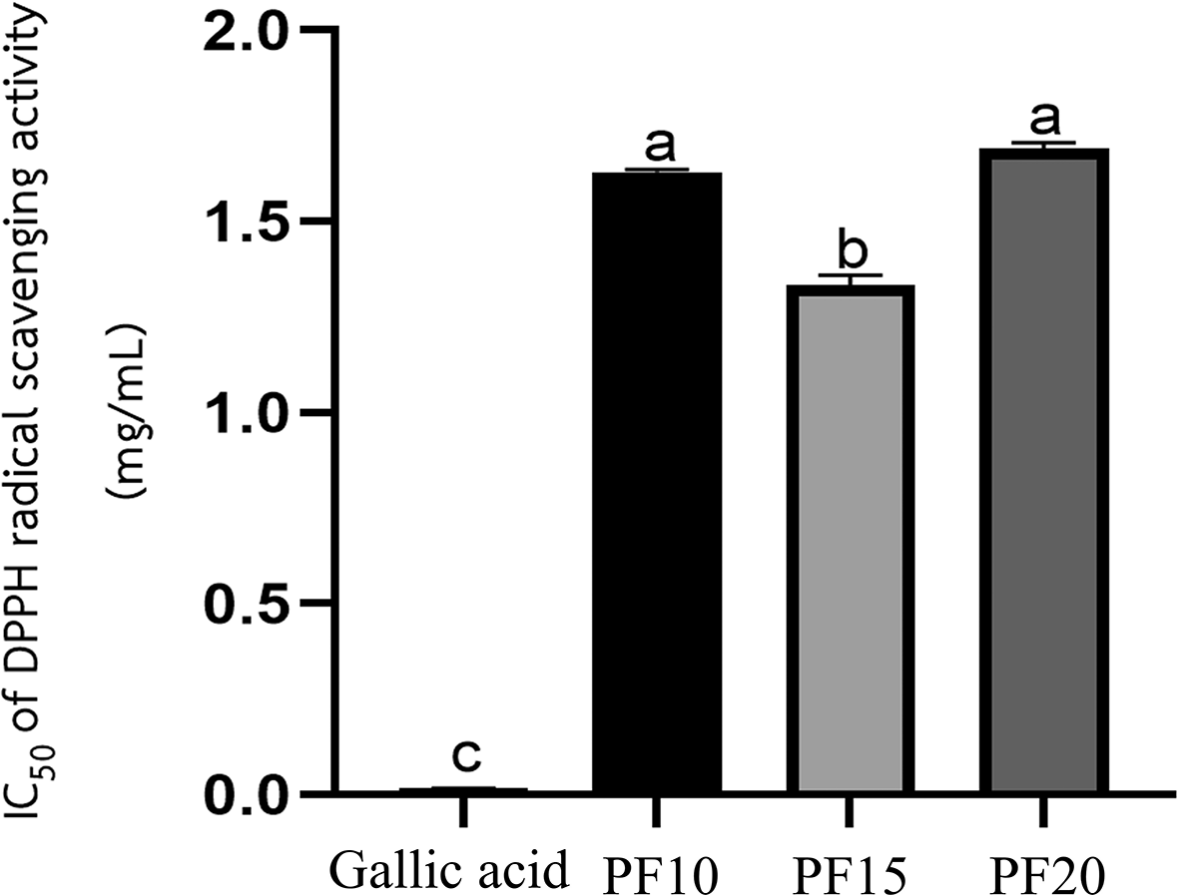
IC _50_ values for DPPH radical scavenging activity of *P. amboinicus* leaf extracts (PF10, PF15, PF20).

The *Plectranthus amboinicus* leaf extract obtained under optimized conditions (PF15) demonstrated significant antioxidant activity, as assessed using the DPPH radical scavenging assay. The extract exhibited a markedly low IC _50_ value, indicating a strong ability to neutralize free radicals. Among the three extraction durations tested—10, 15, and 20 minutes—the PF15 extract showed the most potent antioxidant effect, with a statistically significant difference in IC _50_ values compared to PF10 and PF20 ( *p* < 0.05) ( Figure 7). These findings align with the work of the Ref. 24, who reported the antioxidant potential of *P. amboinicus.* The enhanced activity at 15 minutes suggests that this extraction time enables maximal release and preservation of antioxidant phenolic compounds, including caffeic acid and other flavonoids. This result underscores the importance of extraction time optimization in maximizing the functional properties of herbal extracts. Such antioxidant efficacy supports the potential use of *P. amboinicus* extract in cosmetic formulations targeting oxidative stress-related skin issues, such as aging and environmental damage.

**Antimicrobial activity of PF15**

***Inhibition against skin pathogens***

The antimicrobial potential of PF15 was evaluated using the paper disc diffusion method. At a concentration of 1,000 mg/mL, PF15 exhibited clear zones of inhibition against *S. aureus*, *S. epidermidis*, and *C. acnes.* The diameter of the inhibition zones was significantly larger than those of the negative control (distilled water), indicating strong antibacterial efficacy ( Table 3). These results support the growing evidence that *P. amboinicus* possesses natural antimicrobial compounds effective against common skin pathogens. Its ability to inhibit *C. acnes*, a key contributor to acne development, reinforces its potential application in dermatological formulations such as anti-acne creams and antimicrobial cosmetics. ^23^

**Table 3.** Antibacterial activity of *P. amboinicus* leaf extract (PF15) against selected skin pathogens.

Sample	Inhibition zone diameter (mm)		
	*S. aureus*	*S. eipidermidis*	*C. acnes*
PF5	13.33 ± 0.58	13.33 ± 0.58	15.33 ± 0.58
Ampicillin	27.67 ± 0.58	30.00 ± 1.00	27.67 ± 0.58
Distilled water	-	-	-

Mean ± SD, n = 3.

***Minimum Inhibitory and Bactericidal Concentrations (MIC & MBC)***

The MIC and MBC values of the PF15 extract were determined using a broth microdilution assay. Results indicated that the MIC values against *S. aureus*, *S. epidermidis*, and *C. acnes* were 7.81, 3.91, and 3.91 mg/mL, respectively, while the corresponding MBC values were 31.25, 15.63, and 15.63 mg/mL ( Table 4). These data demonstrate that PF15 not only inhibits bacterial growth but also exhibits bactericidal effects at relatively low concentrations, particularly against *C. acnes*, a clinically relevant acne pathogen. The observed antibacterial potency is consistent with prior reports on the antimicrobial activities of *P. amboinicus.* ^21, 25, 26^ These findings provide strong support for the use of this plant extract in the formulation of herbal-based skin care products designed to prevent or treat bacterial skin infections.

**Table 4.** MIC and MBC of *P. amboinicus* leaf extract (PF15) against selected bacteria.

Sample	MIC (mg/ml)			MBC (mg/ml)		
	*S. aureus*	*S. eipidermidis*	*C. acne*	*S. aureus*	*S. eipidermidis*	*C. acne*
PF15	7.81	3.91	3.91	31.25	15.63	15.63
Ampicillin	0.01	0.01	0.01	0.01	0.01	0.01
Distilled water	-	-	-	-	-	-

**Anti-inflammatory activity of PF15**

***Effect on THP-1 cell viability***

**Figure 8.**
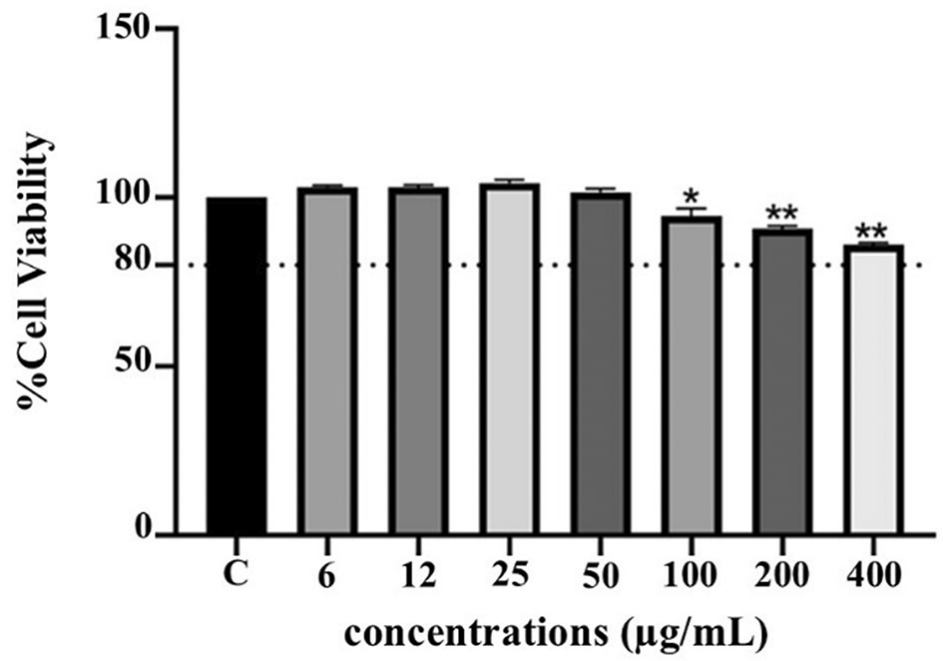
Effect of *P. amboinicus* leaf extract (PF15) on THP-1 cell viability measured using the PrestoBlue ^®^ assay. Data represents the mean ± SD from four independent experiments. * *p* < 0.01 and ** *p* < 0.001 compared to control (C) group.

The cytotoxicity of PF15 was assessed in human THP-1 monocytes using the PrestoBlue ^®^ viability assay. As illustrated in Figure 8, PF15 at concentrations ranging from 6 to 50 μg/mL did not significantly reduce cell viability compared to the untreated control group. Cell viability remained above 80% at all tested concentrations, indicating that PF15 was non-cytotoxic under the experimental conditions. A statistically significant increase in viability was observed at certain concentrations ( *p* < 0.01 and *p* < 0.001), suggesting a potential dose-dependent proliferative or protective effect on THP-1 cells.

***Modulation of cytokine secretion in LPS-stimulated THP-1 macrophages***

**Figure 9.**
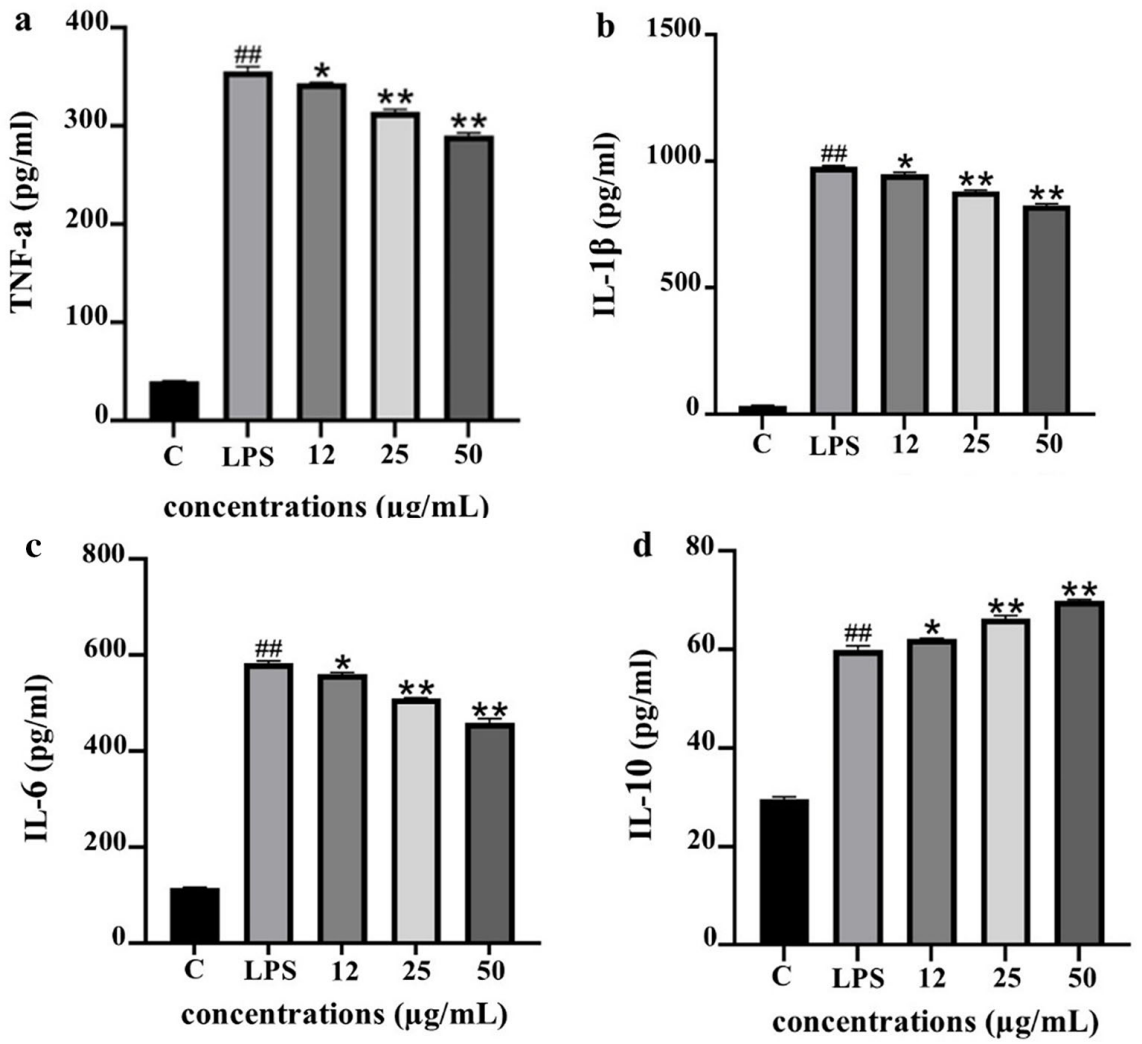
Effects of PF15 on cytokine secretion in LPS-stimulated THP-1 macrophages. ELISA was used to quantify TNF-α (a), IL-1β (b), IL-6 (c), and IL-10 (d). Data are expressed as mean ± SD (n = 3). ## *p* < 0.001 vs. control (C); * *p* < 0.05, ** *p* < 0.001 vs. LPS group.

To evaluate the anti-inflammatory potential of PF15, the levels of pro- and anti-inflammatory cytokines were quantified in lipopolysaccharide (LPS)-stimulated THP-1 macrophages following treatment with PF15 at concentrations of 12, 25, and 50 μg/mL. LPS stimulation significantly increased the secretion of TNF-α, IL-1β, and IL-6 compared to the untreated control ( ^##^ *p* < 0.001). However, treatment with PF15 significantly reduced the levels of these pro-inflammatory cytokines in a dose-dependent manner (* *p* < 0.05 and ** *p* < 0.001), as shown in Figure 9. In contrast, the level of IL-10, a key anti-inflammatory cytokine, was significantly upregulated upon PF15 treatment, suggesting an immunomodulatory shift favoring resolution of inflammation. These results demonstrate that PF15 effectively suppresses pro-inflammatory signaling while enhancing anti-inflammatory responses in macrophages.

**Figure 10.**
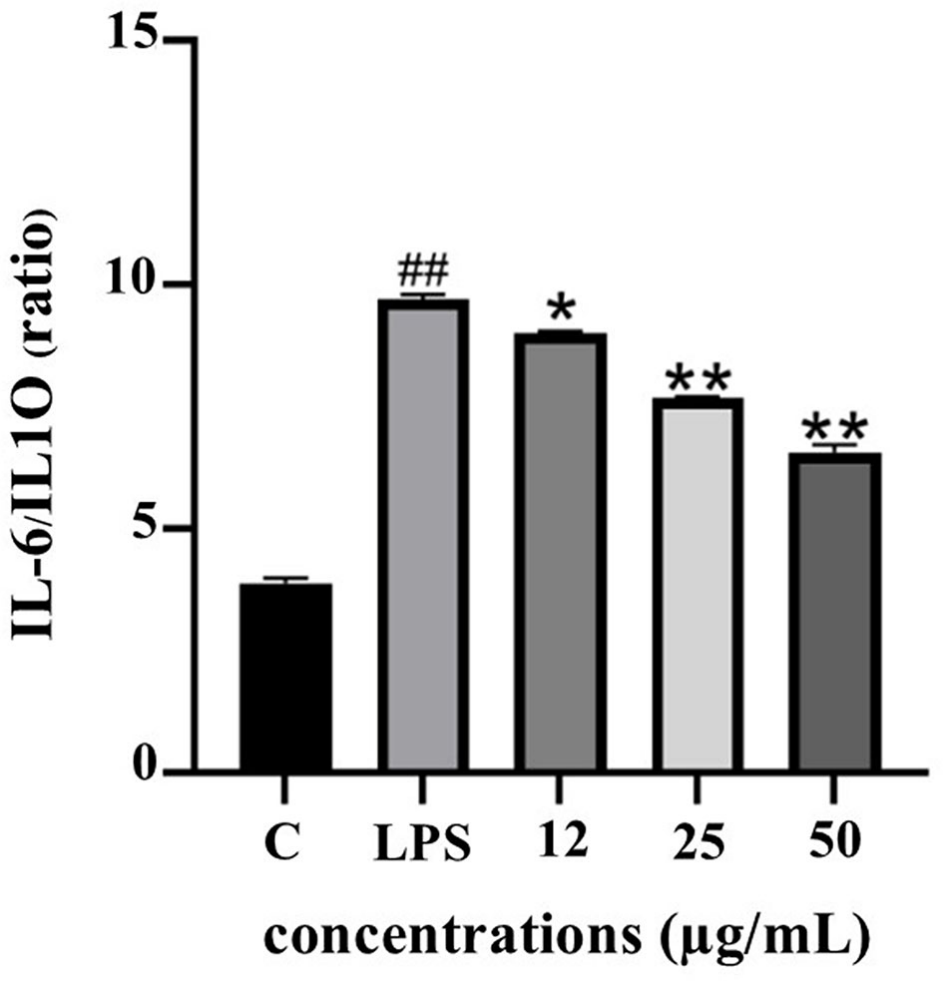
Effect of PF15 on IL-6/IL-10 ratio in LPS-stimulated THP-1 macrophages. Values represent mean ± SD from three independent experiments. ## *p* < 0.001 vs. control; * *p* < 0.01 and ** *p* < 0.001 vs. LPS group.

The IL-6/IL-10 ratio serves as a reliable biomarker for evaluating the balance between pro- and anti-inflammatory responses. As depicted in Figure 10, LPS stimulation significantly elevated the IL-6/IL-10 ratio, indicating a dominant pro-inflammatory state. Treatment with PF15 significantly reduced this ratio in a dose-dependent manner, suggesting a shift toward an anti-inflammatory phenotype and restoration of immune homeostasis.

The results of this study provide robust evidence that *P. amboinicus* leaf extract (PF15) exhibits significant anti-inflammatory activity through multiple mechanisms. The extract not only suppressed key pro-inflammatory cytokines (TNF-α, IL-1β, IL-6) but also enhanced the production of IL-10, a cytokine critical for immune regulation and inflammation resolution. These findings are consistent with previous studies on plant-derived bioactive compounds exhibiting immunomodulatory effects in macrophage models. ^27, 28^ The observed inhibition of TNF-α and IL-6 aligns with established mechanisms of anti-inflammatory plant compounds that interfere with Toll-like receptor (TLR)-mediated signaling pathways. ^29^ Suppression of IL-6 is particularly noteworthy, as its overexpression is implicated in the pathogenesis of chronic inflammatory diseases. ^30^ The upregulation of IL-10 further supports the role of PF15 in promoting an anti-inflammatory environment, a mechanism also observed in other herbal interventions. ^31, 32^ Importantly, the decrease in the IL-6/IL-10 ratio suggests a rebalancing of immune responses, indicative of improved inflammatory resolution. ^33^ Similar regulatory effects have been reported in studies involving flavonoids and polyphenols, ^16, 17^ highlighting the potential of PF15 as a natural anti-inflammatory agent.

### Formulation and development of a topical product containing PF15

**Prototype cream formulation and evaluation**

**Figure 11.**
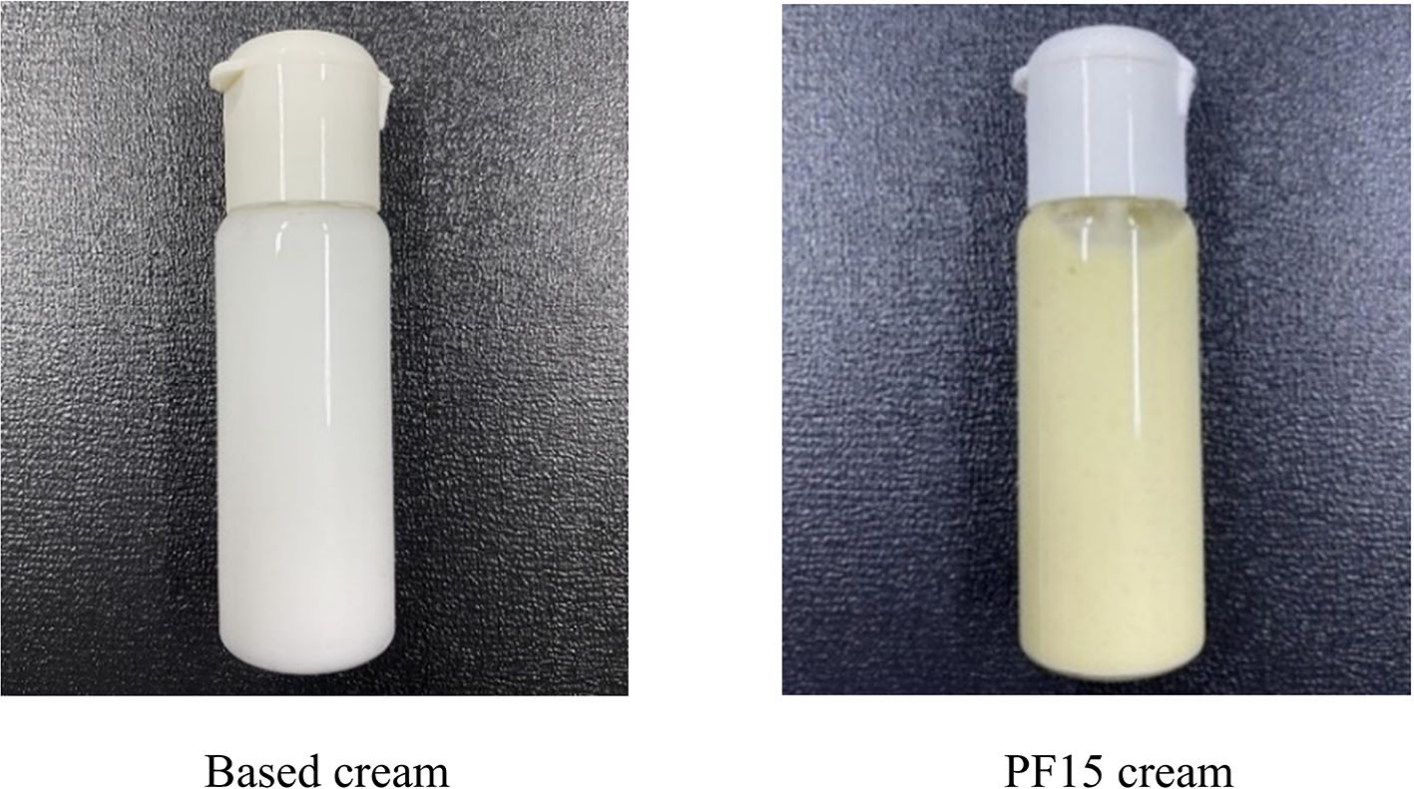
Cream formulation containing PF15 compared to based cream.

A topical cream was formulated using PF15 and compared against a control formulation lacking the extract ( Figure 11). The extract-based cream displayed a light green color, smooth consistency, and favorable spreadability. It emitted a mild, herbal-minty scent, characteristic of the extract, and exhibited no signs of phase separation, sedimentation, or instability ( Table 5). The measured pH of 5.5 aligns with the optimal range for topical applications and skin compatibility. ^34^ These physical and organoleptic properties indicate the successful incorporation of *P. amboinicus* extract into a stable cosmetic matrix with potential applications in acne treatment and skin care product development. ^35^

**Table 5.** Physical properties of topical cream formulations.

Product	Texture	Color	Phase separation	Fragrance	pH
Based cream	Well-dispersed	White	None	Cream-specific	5.5
PF15 cream	Well-dispersed	Light green	None	Minty/herbal	5.5

**Accelerated stability assessment of PF15 cream**

The PF15 cream formulation was subjected to six thermal cycles, involving alternating storage at 4 ± 1 °C and 45 ± 1 °C, as a preliminary stress test to evaluate its stability under accelerated conditions. The cream maintained a consistent texture, color, and fragrance across all cycles, with no evidence of phase separation or degradation. The pH remained stable at 5.5 throughout the testing period, confirming the formulation’s stability under extreme storage conditions. ^36– 38^

**Antibacterial efficacy of formulated PF15 cream**

**Figure 12.**
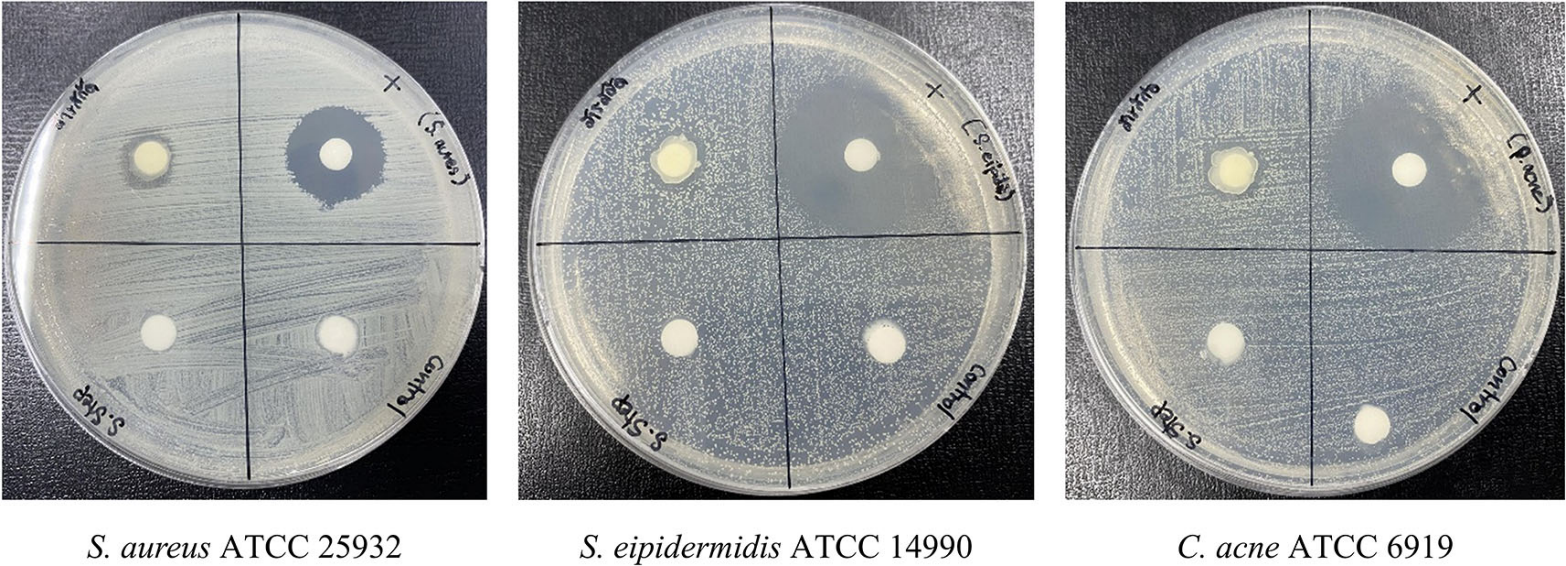
Antibacterial activity of PF15 cream post-stability testing, assessed using the paper disc diffusion method.

Following accelerated stability testing, the PF15 cream retained its antibacterial activity against *S. aureus*, *S. epidermidis*, and *C. acnes.* The inhibition zones measured 11± 1.00 mm, 12± 0.82 mm, and 12± 0.82 mm (Mean ± SD, n = 3) respectively ( Figure 12), confirming that the antimicrobial properties of the extract were not compromised during storage. These results support its efficacy as a topical agent targeting acne-associated pathogens. ^39, 40^

**Microbial contamination assessment**

Microbiological testing of the PF15 cream was conducted in compliance with the Thai Cosmetics standard and ^41^ The cream was assessed for aerobic plate count (APC), yeast and mold contamination, and the presence of *Escherichia coli*, *Staphylococcus aureus*, and *Salmonella* spp. All results were within the acceptable limits, confirming the microbiological safety of the final product ( Table 6).

**Table 6.** Microbial contamination test results of PF15 cream.

Microbial group	Test result	Unit	Standard limit
Aerobic plate count	Not detected	CFU/g	<10 ^3^ CFU/g
Yeasts and molds	Not detected	CFU/g	<10 ^3^ CFU/g
*E. coli*	<3	MPN/g	<10 MPN/g
*S. aureus*	Not detected	CFU/g	Not detected
*Salmonella* spp.	Not detected	in 25 g	Not detected

These findings confirm that the PF15 cream meets safety standards for cosmetic use, is free from pathogenic microorganisms, and is suitable for topical applications. Safety evaluations based on FDA guidelines—including cytotoxicity and microbial contamination assessments—indicate that the formulation is safe for consumer use. This aligns with previous safety studies of *P. amboinicus* in cosmetic applications. ^42^ The successful formulation of a stable, effective, and safe cream containing *P. amboinicus* leaf extract (PF15) demonstrates the practical potential of this plant as a bioactive ingredient in dermatological and cosmeceutical products. The extract retained its biological properties post-formulation and post-stability testing, making it a promising candidate for anti-acne, antimicrobial, and anti-inflammatory skincare applications.

Conventional extraction methods, such as maceration, percolation, decoction, and infusion, primarily rely on the diffusion of bioactive compounds from solid matrices into liquid solvents. These techniques typically require multiple extraction cycles, elevated temperatures, and/or mechanical agitation to enhance mass transfer efficiency, which consequently results in prolonged processing times and high energy consumption. To overcome these limitations, increasing attention has been directed toward the development and application of green extraction technologies that aim to reduce the environmental footprint of extraction processes while ensuring the safety and quality of the final extracts for human consumption. Among these emerging techniques are Microwave-Assisted Extraction (MAE), Supercritical Fluid Extraction (SFE), Pressurized Liquid Extraction (PLE), Ultrasonic-Assisted Extraction (UAE), Enzyme-Assisted Extraction (EAE), and Pulsed Electric Field Extraction (PEFE). These methods are gaining widespread adoption in the fields of food and plant analysis due to their significant advantages, including reduced extraction time, lower consumption of hazardous organic solvents, improved extraction yields, enhanced energy efficiency, and increased reproducibility through automation. The selection of an appropriate extraction method is influenced by various factors, including the physicochemical properties of the target compounds and the specific performance requirements of the extraction process. ^43^ Notably, MAE has been shown to require comparatively smaller solvent volumes, especially when utilizing green solvents such as water or ethanol, thereby decreasing both environmental impact and downstream processing costs. Furthermore, the operational parameters of MAE, such as microwave power, extraction time, and solvent-to-solid ratio, can be precisely controlled and automated, contributing to the method’s reliability and scalability in both research and industrial settings.

## Conclusion

This study provides compelling evidence that *P. amboinicus* leaf extract, obtained through microwave-assisted extraction using 50% ethanol and stabilized via freeze-drying, is a rich source of bioactive compounds—particularly caffeic acid and total phenolics. The extract exhibited multifaceted biological activities, including potent antioxidant capacity, broad-spectrum antimicrobial effects against *S. aureus*, *S. epidermidis*, and *C. acnes*, and significant anti-inflammatory properties through the downregulation of pro-inflammatory cytokines (TNF-α, IL-1β, and IL-6). These findings support the potential use of *P. amboinicus* extract as a natural anti-inflammatory and antimicrobial agent suitable for incorporation into cosmetic and dermatological formulations. Importantly, the extract demonstrated excellent physicochemical stability and microbiological safety when formulated into a topical cream, retaining both efficacy and formulation integrity after accelerated stability testing.

Further investigations—including in vivo efficacy studies and mechanistic analyses at the molecular level—are recommended to confirm the therapeutic relevance and elucidate the pathways involved. Overall, this research highlights the strong potential of *P. amboinicus* as a multifunctional, safe, and stable active ingredient in the development of innovative skincare products, particularly those targeting inflammation, acne, and oxidative stress-related skin conditions.

## Institutional review board

Ethical approval and consent were not required.

## AI use disclosure

In accordance with the Taylor & Francis AI Policy, generative AI tools (ChatGPT 4.0, OpenAI) were used solely for language editing and phrasing suggestions. All usage was conducted under full human supervision.

## Data Availability

The data that support the findings of this study are openly available from Zenodo at: https://zenodo.org/record/15796465 (doi: 10.5281/zenodo.15796465). This dataset was made available under the Creative Commons Attribution 4.0 International (CC BY 4.0) license. ^44^
