# Supplementary material for: Bioactive Effects of Plectranthus amboinicus Extract Using Microwave Techniques and Its Value Addition in Cosmeceutical Products
Source: F1000Res. 2026 Jan 24;14:796. Originally published 2025 Aug 18. [Version 3] doi: 10.12688/f1000research.165030.3 (PMC12828258; doi:10.12688/f1000research.165030.3)
Supplement: Peer review history

# Reviewer report: Reviewer response for version 3

**DOI:** 10.5256/f1000research.195380.r443293

**Published:** 2026-02-05

**Authors:** Md Suzauddula Suzauddula

**Affiliations:** 1 Kansas State University, Manhattan, KS, USA

Reviewer Comments

**Title**

1. The title is generally acceptable and reflects the scope of the study.
2. The manuscript contains typographical symbols likely introduced by AI-assisted writing. Please replace the em dash **“—”** with a hyphen **“-”** throughout the manuscript.
  For example:
*“Two drying techniques—tray drying and freeze-drying—were compared…”*
  should be revised to:
*“Two drying techniques-tray drying and freeze-drying-were compared…”*

**Introduction**

1. Please add appropriate citations to support the statement regarding the **adverse effects associated with synthetic chemicals used in cosmetic formulations**, including allergic reactions, skin irritation, and potential long-term health risks.
2. Please replace all instances of **“—”** with **“-”** in the Introduction section.
3. The following statement requires supporting references:
*“Plectranthus amboinicus, a perennial herb indigenous to Southeast Asia, has been widely utilized in traditional culinary practices and herbal medicine…”*
  Please add citations supporting its **ethnobotanical use, phytochemical composition, and reported antioxidant, antimicrobial, and anti-inflammatory properties**.
4. Please revise the writing style to avoid repetitive phrasing.
  For example, instead of:
*“A recent study by Ref. 6 demonstrated the antimicrobial efficacy…”*
  use:
*“A recent study demonstrated the antimicrobial efficacy…”*
5. Please avoid unnecessary capitalization within sentences. For example, **“Soxhlet”** should not be capitalized unless it begins a sentence.
6. The Introduction would benefit from a brief discussion of the **enzymes measured in this study**, including their **biological relevance and importance in cosmeceutical applications**. The authors are encouraged to cite **recent literature** to contextualize their selection.
  Additionally, please **justify the choice of specific microbiota** used in this research and explain their relevance to the study objectives.

**Methods**

1. For method validation, please cite the **original or reference study** from which the compound extraction protocol was adopted or modified.
2. If the chemical characterization method was adapted from previous work, please **cite the relevant source** and clarify any modifications made.
3. **Cytokine measurement by ELISA:** Please elaborate on the experimental procedure, including kit details and detection principles, and provide a **proper citation** (e.g., manufacturer’s protocol or published reference).
4. **Cream formulation with PF15:** Please validate the formulation method with an appropriate citation from prior literature or formulation guidelines.
5. Please briefly explain the **disc diffusion method**, including its principle and purpose, with a supporting reference.
6. Table 2 requires justification for the **specific percentages of ingredients used**. Please explain the rationale for the chosen formulation composition.
7. For microbial enumeration on Dichloran Rose Bengal Chloramphenicol agar, please provide additional methodological details, including:

- Dilution factors and number of dilution steps
- Diluent used
- Incubation temperature and ambient growth conditions
- Initial microbial load

1. **Escherichia coli detection:** Please include incubation time, temperature, initial microbial concentration, and a brief explanation of the detection method with citation.
2. **Staphylococcus aureus detection:** Please include incubation time, temperature, initial microbial concentration, and a brief explanation of the method with citation.
3. **Salmonella spp. detection:** The absence of dilution requires justification. Please extend the protocol description and include a supporting reference.
4. Please clearly define and classify all formulations/abbreviations used (e.g., **PT10, PT15, PT20, PF10, PF15, PF20**) when first introduced.

**Results**

1. In **Figure 2**, no clear significant difference is observable between tray-dried (TD) and freeze-dried (FD) samples at 15 minutes. Please recheck the data and statistical analysis.
2. In **Figure 4**, no statistical indicators are shown; however, the authors state that caffeic acid content in freeze-dried extracts was significantly higher. Please clarify:

- Where the statistical analysis is presented
- Number of replicates used
- How significance was determined

1. **Figure 5:** Please rearrange the bars so that **PT10 and PF10**, **PT15 and PF15**, and **PT20 and PF20** are displayed side by side for easier comparison.
2. The Results section would benefit from the inclusion of **recent citations and comparative discussion** with similar studies to contextualize the findings. At present, interpretation is limited, and a **major revision** is recommended to strengthen scientific relevance.

Is the work clearly and accurately presented and does it cite the current literature?

Partly

If applicable, is the statistical analysis and its interpretation appropriate?

I cannot comment. A qualified statistician is required.

Are all the source data underlying the results available to ensure full reproducibility?

Yes

Is the study design appropriate and is the work technically sound?

No

Are the conclusions drawn adequately supported by the results?

Partly

Are sufficient details of methods and analysis provided to allow replication by others?

No

Reviewer Expertise:

Bioactive compounds, microbial study, cancer study, and GMO food.

I confirm that I have read this submission and believe that I have an appropriate level of expertise to confirm that it is of an acceptable scientific standard, however I have significant reservations, as outlined above.

# Reviewer report: Reviewer response for version 2

**DOI:** 10.5256/f1000research.189257.r445681

**Published:** 2026-01-22

**Authors:** Supenya Chittapun

**Affiliations:** 1 Thammasat University, Khlong Nueng, Thailand

The manuscript entitled “Bioactive Effects of *Plectranthus amboinicus* Extract Using Microwave Technique and Its Value Addition in Cosmeceutical Products” presents an interesting study combining microwave-assisted extraction, biological activity evaluation, and preliminary product formulation.

 The topic is relevant to the fields of natural products, green extraction technologies, and cosmeceutical development. However, several methodological, reporting, and interpretative issues need to be addressed to improve scientific rigor, reproducibility, and clarity. Many of these points relate to standard requirements for botanical studies, extraction methodology, statistical analysis, and biological interpretation. The following comments are provided section by section, aligned with the manuscript structure, for the authors’ careful consideration.

**Title and Abstract:**

 -The scientific name of the plant should follow botanical nomenclature rules. At first mention, the full scientific name including the authority should be provided (e.g., *Plectranthus amboinicus* (Lour.) Spreng.).

 -Claims made in the abstract regarding extraction efficiency, bioactivity, and formulation performance should be supported by clear methodological descriptions and appropriate statistical analyses in the main text.

**Introduction:**

 -At the first occurrence, the scientific name of the plant should be written in full with the author citation. Subsequent mentions should use the abbreviated genus name (e.g., *P. amboinicus*). If more than one species within the same genus is mentioned, care should be taken to avoid ambiguity.

 -The statement “Recently, microwave-assisted extraction using 50% ethanol has emerged as a promising alternative” requires a clear and specific citation. Please identify the primary literature supporting this claim.

 -The introduction would benefit from a clearer justification for focusing on drying methods (tray drying vs. freeze-drying) in addition to extraction time, and how these factors are expected to influence chemical composition and biological activity.

**Materials and Methods**

***Plant Material and Sample Preparation***

 The source of the plant material should be described in greater detail. Please specify:

 -Location of collection (area, district, province, country)

 -Date or season of collection

 -Plant age or growth stage (if available)

 -Plant part used

 -Whether a voucher specimen was prepared and deposited, including voucher number and herbarium

 This information is essential because phytochemical composition can vary with plant age, season, and geographical location.

***Extraction and Drying Procedures***

 -The number of experimental replicates should be clearly stated for all extraction experiments, including each extraction time and drying method.

 -Microwave-assisted extraction may generate heat depending on extraction time and power. Please clarify whether the actual temperature during extraction was monitored and recorded, and if so, how temperature variation was controlled.

 -Please explain how the absence of residual ethanol in the dried extracts was confirmed prior to biological testing, as residual solvent may influence antimicrobial and cell-based assay results.

 -Abbreviations such as PT10, PT15, PT20, PF10, PF15, and PF20 should be clearly defined at their first occurrence in the text.

***Instrumentation and Chemicals***

 -All instruments used should be reported with full details, including brand name, model, manufacturer, and country of origin, to ensure reproducibility.

***Statistical Analysis***

 - Given that the study involves two independent factors: extraction time and drying method, the use of two-way ANOVA would be more appropriate than one-way ANOVA. This would allow evaluation of the main effect of extraction time, the main effect of drying method and potential interaction effects between these two factors.

***Microbiological and Cell-Based Assays***

 -Since microbial strains were used in this study, please clarify whether approval or exemption from an institutional biosafety committee was obtained, or explicitly state the biosafety level under which the experiments were conducted.

 -The rationale for selecting specific extract concentrations for anti-inflammatory assays should be explained, particularly given that cytotoxicity was not observed at much higher concentrations.

**Results**

 -All figures should include clearly labeled x- and y-axes with appropriate units.

 -Statistical methods used for data analysis should be stated in each figure legend, including type of statistical test, significance levels, explanation of superscript letters or symbols used to indicate statistical differences.

 -For Table 3, statistical analysis should be included to support comparisons among treatments.

 -For Table 4, results should be reported as mean ± standard deviation, based on clearly stated replicate numbers.

 -For Figure 8, the claim of a dose-dependent effect should be supported by appropriate statistical or trend analysis, rather than descriptive observation alone.

 -For Figure 9, the authors should expand the discussion to explain the possible biological mechanisms underlying the observed anti-inflammatory effects, rather than only describing changes in cytokine levels.

**Discussion**

 -As crude extracts were used for biological testing, the authors should discuss which classes of compounds (e.g., caffeic acid, other phenolics, or synergistic mixtures) are most likely responsible for the observed activities.

 -The discussion should avoid overinterpretation of results and clearly distinguish between experimentally demonstrated effects and proposed mechanisms.

**Formulation Section**

 -The rationale for selecting a water-in-oil cream base for an anti-acne application should be explained, as oil-rich formulations may influence skin compatibility and acne-related outcomes.

**Data Presentation and Formatting**

 -Numerical data throughout the manuscript should be reported with consistent decimal places, reflecting the precision of the analytical methods and associated standard deviations.

 -Please carefully revise the manuscript for language consistency, grammar, and formatting, particularly the use of hyphens and compound terms (e.g., “anti-inflammatory”).

 In summary, the manuscript addresses a relevant and timely topic; however, substantial revisions are required to improve methodological transparency, statistical rigor, and interpretative depth. Addressing the points outlined above will significantly enhance the scientific quality, reproducibility, and impact of the study. I encourage the authors to carefully revise the manuscript accordingly.

Is the work clearly and accurately presented and does it cite the current literature?

Yes

If applicable, is the statistical analysis and its interpretation appropriate?

Partly

Are all the source data underlying the results available to ensure full reproducibility?

Partly

Is the study design appropriate and is the work technically sound?

Yes

Are the conclusions drawn adequately supported by the results?

Yes

Are sufficient details of methods and analysis provided to allow replication by others?

Partly

Reviewer Expertise:

Algal biotechnoloogy

I confirm that I have read this submission and believe that I have an appropriate level of expertise to confirm that it is of an acceptable scientific standard, however I have significant reservations, as outlined above.

# Reviewer report: Reviewer response for version 2

**DOI:** 10.5256/f1000research.189257.r443296

**Published:** 2026-01-14

**Authors:** Nguyen Thanh Triet

**Affiliations:** 1 University of Medicine and Pharmacy at Ho Chi Minh City, Ho Chi Minh City, Vietnam

1. The authors should mention who identified the plant sample? When was the sample collected? What is the name of plant voucher specimen?

 2. The authors should mention in detail how they established the optimization of extraction method?

 3. The authors need to explain why they used caffeic acid as a marker to evaluate the quality of the extract and optimize the extraction method?

 4. Bioactivity assays need to be described in detail, especially which indicators were used to evaluate the results and the calculation formulas.

 5. Did the authors see other peaks after 15 minute-run in HPLC chromatogram?

Is the work clearly and accurately presented and does it cite the current literature?

Yes

If applicable, is the statistical analysis and its interpretation appropriate?

Yes

Are all the source data underlying the results available to ensure full reproducibility?

Yes

Is the study design appropriate and is the work technically sound?

Yes

Are the conclusions drawn adequately supported by the results?

Yes

Are sufficient details of methods and analysis provided to allow replication by others?

Yes

Reviewer Expertise:

pharmacognosy, traditional pharmacy, ethnopharmacology, botanical classification, botanical anatomy, phytochemistry

I confirm that I have read this submission and believe that I have an appropriate level of expertise to confirm that it is of an acceptable scientific standard, however I have significant reservations, as outlined above.

# Response

**Published:** 2026-01-16

**Authors:** Doungporn Amornlerdpison

**Affiliations:** Maejo University, Nong Han, Chiang Mai, Thailand

**Response to Reviewer’s Comments**

 We sincerely thank the reviewer for the constructive and insightful comments. Our responses and corresponding revisions are provided below. The changes have been made as highlighted in “red font” in revised manuscript (Version 3).

 1. The authors should mention who identified the plant sample? When was the sample collected? What is the name of plant voucher specimen?

 The plant material ( *Plectranthus amboinicus) *was taxonomically identified and authenticated by Assoc. Prof. Dr. Yuwalee Unpaprom, a botanist at the Center of Excellence in Agricultural Innovation for Graduate Entrepreneur (Agri Inno), Maejo University, Chiang Mai, Thailand. Fresh leaves were collected in April 2025 from the Maejo area, San Sai District, Chiang Mai Province, Thailand. A voucher specimen has been prepared and deposited at Agri Inno under voucher number Agri Inno- 2574. This information has now been added to the Materials and Methods section of the revised manuscript.

 2. The authors should mention in detail how they established the optimization of extraction method?

 We acknowledge that a formal optimization design, such as Response Surface Methodology or factorial design, was not conducted in this study. Accordingly, the manuscript has been revised to avoid the use of the term “optimized.” Instead, the extraction conditions were **systematically evaluated and screened** based on microwave-assisted extraction times (5, 10, and 15 minutes) in combination with two drying methods (tray drying and freeze-drying). The selection of the PF15 condition was based on comparative outcomes including extraction yield, total phenolic content, caffeic acid content, and antioxidant activity. The revised manuscript now clearly reflects this screening-based approach.

 3. The authors need to explain why they used caffeic acid as a marker to evaluate the quality of the extract and optimize the extraction method?

 Caffeic acid was selected as a marker compound because it is a well-documented major phenolic constituent of *P. amboinicus* and is closely associated with its biological activities. Preliminary HPLC screening showed that caffeic acid was the most prominent and consistently detectable peak. Screening with standards of other phenolics, including chlorogenic acid and quercetin, did not show detectable peaks under the applied conditions. Therefore, caffeic acid was used as the representative marker for extract evaluation.

 4. Bioactivity assays need to be described in detail, especially which indicators were used to evaluate the results and the calculation formulas.

 Bioactivity assays are now described in greater detail in the revised manuscript, including the indicators used and calculation approaches. The concentration of PF15 incorporated into the cream formulation was selected based on antimicrobial efficacy and formulation feasibility. The minimum inhibitory concentration (MIC) of PF15 against *Staphylococcus aureus* was determined to be **7.8 mg/mL**. To ensure effective antimicrobial performance in a topical dosage form, PF15 was incorporated at **0.8% w/w**, corresponding to approximately **8 mg/g of formulation**, consistent with the MIC obtained from in vitro testing.



 This concentration was selected to ensure sufficient antimicrobial activity while maintaining formulation stability, acceptable sensory properties, and suitability for topical application. Antimicrobial testing of the finished PF15 cream demonstrated inhibition zones of 11–13 mm against selected skin pathogens, confirming that the bioactivity of PF15 was retained after emulsification and thermal processing. Although a slight reduction in inhibition zone diameter compared with the crude extract (13–15 mm) was observed, the extract remained functionally active within the cream matrix.

 5. Did the authors see other peaks after 15 minute-run in HPLC chromatogram?

 As shown in Figures 3 and 4, no additional significant peaks were observed within the 15-minute HPLC run. The chromatographic analysis was designed as a targeted method focusing on the retention time of caffeic acid, which was clearly detected and consistently resolved under the applied analytical conditions. Therefore, the analytical window was limited to 15 minutes to enable reliable and reproducible comparison of extraction conditions using caffeic acid as the representative marker compound.

# Reviewer report: Reviewer response for version 1

**DOI:** 10.5256/f1000research.181624.r413222

**Published:** 2025-09-27

**Authors:** Nuntawat Khat-Udomkiri

**Affiliations:** 1 Mae Fah Luang University, Mueang Chiang Rai, Chiang Rai, Thailand

Thank you for the opportunity to review this manuscript. The study of microwave-assisted extraction (MAE) from Plectranthus amboinicus for cosmeceutical use is topical and fits the journal scope, but the manuscript in its current form lacks novelty, methodological detail, and sufficient experimental rigor. Below I list the main weaknesses and give concrete, actionable recommendations to improve clarity, reproducibility and scientific impact.

 1. You must cite and discuss prior MAE work on P. amboinicus (e.g., the paper you identified: DOI: 10.3303/CET1972067). Clearly state how your study differs (new endpoints, different solvent system, biological assays, formulation work, scale-up considerations, etc.). Right now the manuscript reads like a repeat of existing work.

 2. Do not claim you “optimized” extraction conditions unless a formal design of experiments (e.g., RSM, factorial) or an explicit optimization protocol was applied. If you did not run an optimization design, rephrase to “screened”, “evaluated” or “investigated”.

 3. Temperature is critical for MAE outcomes and must be reported for every condition. Report temperature profiles for all MAE runs.

 4. Explain the basis for selecting caffeic acid as the principal marker. Was it the most abundant compound in the extract, the most bioactive, or selected for analytical convenience?

 5. Natural extracts are mixtures — identify and quantify multiple major phenolics, not only one. Provide HPLC chromatograms and quantification for at least the top 3–5 phenolic compounds.

 6. Provide a full cream formulation (all ingredients, % w/w), the exact amount of extract incorporated, and the rationale for the selected concentration(s). 

 7. You state extract was included during emulsification: explain and justify this choice. Phenolic compounds is heat-sensitive; if used in a heated phase, show data proving it remains active after processing. 

 8. You cannot assert “no degradation” based on visual inspection alone. Provide objective stability data: HPLC profiles (initial vs after processing and after stability cycles), TPC, and at least one chemical marker quantified before/after heating and after storage cycles. Replace subjective color assessments with ΔE measurements and report ΔE for color change.

 9. DPPH must include a positive control (e.g., Trolox or ascorbic acid) and report results as IC₅₀ or mg Trolox equivalent/g for comparability.

 10. Antimicrobial claims cannot use “broad-spectrum” without testing a representative panel. 

 11. Key MAE factors were omitted (type of solvent, solvent concentration, power, temperature, time, solid:liquid ratio). If you want to claim optimization, perform a DOE (factorial or RSM). Otherwise clearly present the work as a screening study.

 12. Rephrase or remove any absolute claims (e.g., “broad-spectrum”, “no degradation”) unless supported by data.

 13. Reword the heat-cycling sentence: heating–cooling is a preliminary shelf-stress test, not a full simulation of long-term stability. Use careful language.

 14. Update and expand references: several cited works are dated — include recent MAE and cosmeceutical formulation literature.

 15. Drying methods, whether heat-based (e.g., tray drying) or non-thermal (e.g., freeze-drying), are well-documented for their influence on phenolic compound stability and retention. Please clarify the rationale for selecting this factor as the focus of investigation rather than prioritizing the optimization of parameters directly affecting the extraction process.

Is the work clearly and accurately presented and does it cite the current literature?

Yes

If applicable, is the statistical analysis and its interpretation appropriate?

Partly

Are all the source data underlying the results available to ensure full reproducibility?

Partly

Is the study design appropriate and is the work technically sound?

No

Are the conclusions drawn adequately supported by the results?

No

Are sufficient details of methods and analysis provided to allow replication by others?

No

Reviewer Expertise:

Non-conventional extraction. Bioactive compounds, Cosmetics, Cell-culture assays, Formulations

I confirm that I have read this submission and believe that I have an appropriate level of expertise to state that I do not consider it to be of an acceptable scientific standard, for reasons outlined above.

# Response

**Published:** 2025-10-03

**Authors:** Doungporn Amornlerdpison

**Affiliations:** Maejo University, Nong Han, Chiang Mai, Thailand

**Reviewer 2 (Not Approved)**

 1. You must cite and discuss prior MAE work on P. amboinicus (e.g., the paper you identified: DOI: 10.3303/CET1972067). Clearly state how your study differs (new endpoints, different solvent system, biological assays, formulation work, scale-up considerations, etc.). Right now the manuscript reads like a repeat of existing work.

Response: Our study differs by evaluating drying methods (tray vs freeze-drying), biological activities (antimicrobial and anti-inflammatory properties) and formulation into a cream with stability testing.

 2. Do not claim you “optimized” extraction conditions unless a formal design of experiments (e.g., RSM, factorial) or an explicit optimization protocol was applied. If you did not run an optimization design, rephrase to “screened”, “evaluated” or “investigated”.

Response: We agree and have rephrased “optimized” to “evaluated”, Page 2 unless clearly supported by data.

 3. Temperature is critical for MAE outcomes and must be reported for every condition. Report temperature profiles for all MAE runs.

Response: We have added the extraction temperature (40 °C under 450 W microwave power) in the Methods section, Page 3

 4. Explain the basis for selecting caffeic acid as the principal marker. Was it the most abundant compound in the extract, the most bioactive, or selected for analytical convenience?

Response: We selected caffeic acid as a marker because it was the most abundant phenolic identified in preliminary HPLC profiling. This justification has been added, Page 3.

 5. Natural extracts are mixtures — identify and quantify multiple major phenolics, not only one. Provide HPLC chromatograms and quantification for at least the top 3–5 phenolic compounds.

Response: In this study, a representative phenolic compound, caffeic acid was selected as a biomarker for quantification due to its well-documented bioactivity. Furthermore, the use of total phenolic content (TPC) provides a reliable estimation of the overall phenolic load in the extracts. This approach enables holistic evaluation of the extract's functionality while minimizing the complexity, time, and cost associated with full chromatographic profiling. Future work may include expanded HPLC profiling to better understand the full phenolic spectrum and assess potential synergistic effects among individual compounds.

 6. Provide a full cream formulation (all ingredients, % w/w), the exact amount of extract incorporated, and the rationale for the selected concentration(s).

Response: We have revised the section to provide the complete formulation with all ingredients and concentrations (% w/w)

 The formulation was based on the minimum inhibitory concentration (MIC) of PF15, determined to be 7.8 mg/mL against *Staphylococcus aureus*. For a 100 g cream formulation (equivalent to approximately 100 mL), the required amount of extract was calculated as 780 mg, corresponding to 0.78% (w/w) of the total formulation. The 0.8% concentration was selected to ensure antimicrobial efficacy while maintaining formulation stability, Page 5-6.

 7. You state extract was included during emulsification: explain and justify this choice. Phenolic compounds is heat-sensitive; if used in a heated phase, show data proving it remains active after processing.

Response: The PF15 extract was incorporated during the emulsification step to ensure uniform distribution of the bioactive compounds within the cream matrix. Although phenolic compounds are generally considered heat-sensitive, our results demonstrate that the antimicrobial activity of the extract was largely retained after processing. The PF15 extract alone showed inhibition zones of 13–15 mm (Table 3, Page 12) against selected skin pathogens, while the formulated PF cream exhibited inhibition zones of 11–12 mm (Page 16). The slight reduction indicates minor thermal impact; however, the preserved inhibitory activity confirms that the phenolic constituents remained bioactive after emulsification.

 8. You cannot assert “no degradation” based on visual inspection alone. Provide objective stability data: HPLC profiles (initial vs after processing and after stability cycles), TPC, and at least one chemical marker quantified before/after heating and after storage cycles. Replace subjective color assessments with ΔE measurements and report ΔE for color change.

Response: Deleted no degradation.

 We acknowledge the reviewer’s comment regarding the need for objective stability data. In our study, instead of quantifying individual phenolic compounds within the cream formulation, stability was evaluated based on the functional antimicrobial activity of the product. This approach was selected because the cream contains multiple ingredients. Therefore, antimicrobial efficacy against target skin pathogens was used as a practical stability indicator (Page 16), confirming that the bioactive effect of PF15 was retained after processing and throughout the stability cycles.

 9. DPPH must include a positive control (e.g., Trolox or ascorbic acid) and report results as IC₅₀ or mg Trolox equivalent/g for comparability.

Response: We used gallic acid as the positive control, consistent with its use in the phenolic content determination. Figure 7 has been updated to include gallic acid as the control.

 10. Antimicrobial claims cannot use “broad-spectrum” without testing a representative panel.

Response: Deleted broad-spectrum from conclusion.

 11. Key MAE factors were omitted (type of solvent, solvent concentration, power, temperature, time, solid:liquid ratio). If you want to claim optimization, perform a DOE (factorial or RSM). Otherwise clearly present the work as a screening study.

Response: We have now reported solvent type and concentration (50% ethanol), power (450 W), temperature (40 °C), extraction time (10–20 min), and solid–liquid ratio (1:10 v/v) in the Methods section, Page 3.

 12. Rephrase or remove any absolute claims (e.g., “broad-spectrum”, “no degradation”) unless supported by data.

Response: Statements such as “no degradation” and “broad-spectrum” have been rephrased to reflect the data accurately.

 13. Reword the heat-cycling sentence: heating–cooling is a preliminary shelf-stress test, not a full simulation of long-term stability. Use careful language.

Response: We have reworded this as a “preliminary stress test to evaluate its stability under accelerated conditions”, Page 16

 14. Update and expand references: several cited works are dated — include recent MAE and cosmeceutical formulation literature.

Response: We have updated the reference with recent MAE and other green extraction, Page 17-18 References updated: [43]

 15. Drying methods, whether heat-based (e.g., tray drying) or non-thermal (e.g., freeze-drying), are well-documented for their influence on phenolic compound stability and retention. Please clarify the rationale for selecting this factor as the focus of investigation rather than prioritizing the optimization of parameters directly affecting the extraction process.

Response: Our unique focus on drying and extraction methods relevance for industrial-scale processing and use and effective cost for commercial.

 We believe these revisions strengthen the manuscript significantly by clarifying novelty, improving methodological transparency, and ensuring accurate claims. We hope that the revised version now satisfactorily addresses all reviewer concerns and will be considered suitable for approval

# Reviewer report: Reviewer response for version 1

**DOI:** 10.5256/f1000research.181624.r407511

**Published:** 2025-09-15

**Authors:** Jukkarin Srivilai

**Affiliations:** 1 University of Phayao, Phayao, Thailand

The manuscript titled *“Bioactive Effects of Plectranthus amboinicus Extract Using Microwave Technique and Its Value Addition in Cosmeceutical Products”* presents interesting and technically relevant work. However, several issues need to be addressed before indexing. The following comments are provided for the authors’ consideration:

1. Title: The word “Techniques” should be singular (“Technique”).
2. Abstract: In the Methods section, the last sentence should be revised to: *“Anti-inflammatory potential of PF15 ….”*
3. Dosage form: The authors should clearly state the dosage form of the cream throughout the manuscript, or at least specify somewhere that it is an emulsion type.
4. Introduction: In the third paragraph, please verify reference [6] and revise the sentence for better grammatical clarity.
5. Materials and Methods: The description of the 50% ethanol extraction should specify the solvent used (e.g., DI water or another solvent).
6. Chemical analysis (HPLC): The methodology should clarify whether standard compounds were used to construct the calibration curve for determining the relative amount of caffeic acid, or if another approach was applied. Please provide details.
7. Table 1: The column “Mode” should be labeled “Mode of elution.”
8. Abbreviations: Ensure that abbreviations follow the full name consistently (e.g., Minimal Inhibitory Concentration (MIC)). Please check throughout the manuscript.
9. Cream formulation with PF15: Provide more details on the formulation ratio, type of emulsion, and preparation technique.
10. Results and Discussion:

- Verify the unit of yield, as it may be incorrect.
- In Figure 3, the retention time of the caffeic acid standard does not match the retention time mentioned in the Results and Discussion section. Please correct this, as it is a critical point for analysis.

1. Comparative discussion: Expand the discussion to include other extraction techniques or green solvents. You may cite the following articles:

- [Reference 1] 
- [Reference 2] 

1. Scientific names: On page 10, in the antioxidant activity section, the correct scientific name is *P. amboinicus*. Similarly, check all bacterial names and ensure consistency throughout the manuscript.
2. Table 2:

- Use a big letter instead of  “inhibition” to be Inhibition.
- The caption should specify the sample/disc amount (e.g., 5 mg/disc).

Is the work clearly and accurately presented and does it cite the current literature?

Yes

If applicable, is the statistical analysis and its interpretation appropriate?

Yes

Are all the source data underlying the results available to ensure full reproducibility?

Yes

Is the study design appropriate and is the work technically sound?

Yes

Are the conclusions drawn adequately supported by the results?

Yes

Are sufficient details of methods and analysis provided to allow replication by others?

Yes

Reviewer Expertise:

Plant extraction technologies

I confirm that I have read this submission and believe that I have an appropriate level of expertise to confirm that it is of an acceptable scientific standard.

## References

[1] : Deep eutectic-based microemulsion: A multifunctional system for enhancing extraction efficiency, stability and antityrosinase, antioxidant activities of oxyresveratrol from Artocarpus lakoocha Roxb. *Journal of Molecular Liquids* .2025;436: 10.1016/j.molliq.2025.128253 10.1016/j.molliq.2025.128253

[2] : A novel deep eutectic and eutectic-based microemulsion systems for enhanced extraction, stabilization and antioxidant activity of artocarpin, isocyclomorusin, and cycloartocarpin from agricultural byproduct of Artocarpus heterophyllus Lam. *Microchemical Journal* .2025;217: 10.1016/j.microc.2025.114927 10.1016/j.microc.2025.114927

# Response

**Published:** 2025-10-03

**Authors:** Doungporn Amornlerdpison

**Affiliations:** Maejo University, Nong Han, Chiang Mai, Thailand

We would like to sincerely thank the reviewers for the valuable and constructive comments. We have carefully revised the manuscript according to all suggestions, and below we provide a point-by-point response. The changes have been made as highlighted in “red font” in revised manuscript. We hope our revision has improved the paper to a satisfactory level.

**Reviewer 1 (Approved)**

 1. Title: The word “Techniques” should be singular (“Technique”).

             Corrected to “Technique.”

 2. Abstract: In the Methods section, the last sentence should be revised to: “Anti-inflammatory potential of PF15 ….”

 Revised: “Anti-inflammatory potential of PF15 was evaluated in LPS-stimulated THP-1 macrophages.”

 3.Dosage form: The authors should clearly state the dosage form of the cream throughout the manuscript, or at least specify somewhere that it is an emulsion type.

 Stated in Part: Product Development and Evaluation, Page 5 “The extract was incorporated into the cream formulation at a concentration of 0.8%, ensuring antimicrobial efficacy. The aqueous phase containing PF15 was heated to 70 °C, combined with the oil phase, and emulsified for 30 minutes. The cream was then cooled to 35–40 °C, mixed until homogeneous, and stored for further analysis. A topical cream was developed by incorporating the PF15 extract into a water-in-oil emulsion system. The detailed composition of the cream is presented in Table 2.”

 4.Introduction: In the third paragraph, please verify reference [6] and revise the sentence for better grammatical clarity.

 Verified and corrected sentence, Page 2. “A recent study demonstrated the antimicrobial efficacy of P. amboinicus leaf extract against pathogens such as Staphylococcus aureus, Escherichia coli, Pseudomonas aeruginosa, Candida albicans, and the acne-associated bacterium Cutibacterium acnes, thereby reinforcing its potential application in cosmetic formulations. ^6^

 5.Materials and Methods: The description of the 50% ethanol extraction should specify the solvent used (e.g., DI water or another solvent).

             Clarified as ethanol + deionized water, Page 3.

 “The 50% ethanol solution was prepared using a 1:1 mixture of ethanol and deionized water.”

 - Chemical analysis (HPLC): The methodology should clarify whether standard compounds were used to construct the calibration curve for determining the relative amount of caffeic acid, or if another approach was applied. Please provide details.

 Clarified as “The standard of caffeic acid was used to construct the calibration curve.” 

 - Table 1: The column “Mode” should be labeled “Mode of elution.”

 Corrected. Mode of elution

 - Abbreviations: Ensure that abbreviations follow the full name consistently (e.g., Minimal Inhibitory Concentration (MIC)). Please check throughout the manuscript.

 Checked and corrected; Minimal Inhibitory Concentration (MIC)

 - Cream formulation with PF15: Provide more details on the formulation ratio, type of emulsion, and preparation technique.

 Full formulation ratios, emulsion type, and preparation technique added, Page 5-6.

 6.Results and Discussion:

 - Verify the unit of yield, as it may be incorrect.

 Corrected to % w/w dry weight

 - Figure 3, the retention time of the caffeic acid standard does not match the retention time mentioned in the Results and Discussion section. Please correct this, as it is a critical point for analysis.

 Corrected to 7.8 min retention time for caffeic acid.

 7.Comparative discussion: Expand the discussion to include other extraction techniques or green solvents. You may cite the following articles: [Reference 1] [Reference 2]

 Expanded with ultrasound-assisted, pressurized liquid, and other green solvent extraction, Page 17-18 References updated: [43]
